# An analysis of RMNCH data quality on technological applications and methods to improve it

**DOI:** 10.1093/eurpub/ckac130.202

**Published:** 2022-10-25

**Authors:** H Harhini, P Nath Sidh

**Affiliations:** Sahaj Sansthan, Jodhpur, India

## Abstract

**Background:**

Health information management systems (HIMS) are used by most countries to record data on the coverage of reproductive, maternal, newborn and child health services (RMNCH). The HIMS in India manually collects data from primary health care facilities. Low and middle income countries (LMIC) are shifting to technological applications (apps) to improve reporting and real time tracking of RMNCH services. This study found high disparity of data between different sources. Yet governments and other stakeholders continue to use poorly reported data for daily monitoring, review and decision making. This study shares insight on the high degree of data variation, and proposes process and policy changes to improve it.

**Methods:**

To quantify the extent of data variation, critical RMNCH indicators from apps and HIMS are compared for a given geography and time period. Workshops with the primary health workers and their supervisors were conducted to understand the challenges in reporting, based on which solutions to improve the efficiency of apps are proposed.

**Results:**

Preliminary analysis in the state of Madhya Pradesh shows, in the year 2020-2021 live births, neonatal deaths and Infant deaths were 93%, 91% and 71% less respectively on apps as compared to HIMS. Major challenges of reporting on technological applications by primary health workers

- low internet penetration at 31% in Madhya Pradesh
- Lack of mechanisms to ensure user friendly and glitch-free apps
- Poor capacity to understand public health indicators and application generated reports

**Conclusions:**

Online data quality must be governed by policies that focus on implementing mechanisms to analyze and validate data from different sources and remove blockages to quality reporting. Online public health reports need to be demystified by building capacities of primary health workers and their supervisors to use data to reflect on their performance and plan for improvement.

**Key messages:**

- Poor online data will impact real time action to ensure coverage and accountability for low performance.
- Investing in user friendly efficient technology for RMNCH services with strong quality control mechanisms, is the priority for good decision making.

